# Sociodemographic inequities and use of hybrid closed-loop systems associated with obesity in youth with type 1 diabetes

**DOI:** 10.1016/j.diabres.2025.112041

**Published:** 2025-02-09

**Authors:** Svetlana Azova, Lori Laffel, Belinda S. Lennerz, Carter R. Petty, Joseph Wolfsdorf, Erinn T. Rhodes, Katharine Garvey

**Affiliations:** a Division of Endocrinology, Boston Children’s Hospital, 300 Longwood Avenue, Boston, MA 02115, United States; b Department of Pediatrics, Harvard Medical School, 25 Shattuck Street, Boston, MA 02115, United States; c Joslin Diabetes Center, One Joslin Place, Boston, MA 02215, United States; d Biostatistics and Research Design Center, Boston Children’s Hospital, 300 Longwood Avenue, Boston, MA 02115, United States

**Keywords:** Obesity, Pediatric, Type 1 diabetes, Hybrid closed-loop technology, Child Opportunity Index, Insurance

## Abstract

**Aims:**

This study aimed to describe changes over time in rates of overweight and obesity and to identify factors associated with obesity in youth with type 1 diabetes.

**Methods:**

We analyzed data from 7360 diabetes medical visits among 2242 youth with type 1 diabetes for ≥1 year followed at a pediatric, tertiary care, academic medical center between 2018 and 2023. Multivariable generalized estimating equations (GEE) analysis and conditional logistic regression (CLR), where each patient had both control (not obesity) and case (obesity) status, were conducted.

**Results:**

Adjusted annual percentages of patients with obesity increased from 13.8 % in 2018 to 18.2 % in 2023 (*P* = 0.006); rates of overweight did not differ significantly over time. In multivariable GEE analysis, public insurance (*P* = 0.026), lower Child Opportunity Index score (*P* = 0.027), and use of hybridclosed-loop (HCL) systems (*P* = 0.023) were associated with obesity. In CLR, use of continuous glucose monitor and HCL systems and the sum of their effects (*P* = 0.002) were associated with obesity.

**Conclusions:**

This study revealed increasing rates of obesity in children with type 1 diabetes and identified sociodemographic and diabetes care-related factors associated with obesity, highlighting targets for intervention to decrease future risk of cardiovascular complications.

## 1. Introduction

Overall rates of pediatric obesity (defined as body mass index [BMI] ≥ 95th percentile or BMI z-score ≥1.645 for age and sex) have been rising over the past several decades, with recent available data from the Centers for Disease Control and Prevention estimating that 22.4 % of children and adolescents in the United States (US) had obesity as of August 2020 [1]. As of 2021, the prevalence of overweight and obesity among youth across all 50 states ranged from 21.1 % in boys aged 2–4 years up to 45.6 % in adolescent girls aged 15–19 years, with rates forecasted to rise significantly by 2050 [2]. Several studies have also demonstrated high and rising rates of overweight (defined as BMI ≥ 85th to <95th percentile or BMI z-score ≥1.04 to <1.645 for age and sex) and obesity among pediatric patients with type 1 diabetes, both at the time of diagnosis and with increasing disease duration [3–11]. Obesity has, in turn, been associated with increased risk of future cardiovascular complications, potentially contributing to decreased life expectancy of patients with type 1 diabetes, risks recognized by both the American Diabetes Association (ADA) and International Society for Pediatric and Adolescent Diabetes [12,13].

Previous studies have identified several demographic factors, including female sex [4], older age at diagnosis [9], and historically marginalized background [9,14–16], to be associated with obesity in patients with type 1 diabetes. In terms of diabetes management strategies, the Diabetes Control and Complications Trial (DCCT) notably found that intensive insulin therapy was associated with greater weight gain in participants with type 1 diabetes compared with conventional treatment [17–19]. However, many of these studies were performed before the availability of more physiologic insulin analogs associated with reduced hypoglycemia [20] and most before the widespread use of advanced diabetes technologies, specifically hybrid closed-loop (HCL) systems. The more recent advancements in HCL technology have allowed for automated insulin delivery based on glucose trends, resulting in improved overall diabetes control [21–23], reduced risk of hypoglycemia [21,23–26], and decreased disease burden [21–23,25–27]. However, as these systems may be associated with increased flexibility in food intake [28] and delivery of higher insulin doses in response to postprandial glycemic excursions [29], there is concern that this may lead to excessive weight gain.

The aims of this retrospective study were to 1) describe recent differences over time in the percentage of established pediatric patients with type 1 diabetes who met criteria for overweight and obesity and 2) identify sociodemographic and diabetes care-related factors associated with the presence of obesity. Such data may inform future investigational and interventional studies aimed at addressing overweight and obesity in pediatric type 1 diabetes with the potential to mitigate its development and reduce future risk of adult obesity and cardiovascular complications in this population.

## 2. Subjects, materials, and methods

### 2.1. Study design, setting, and selection of participants

This was a retrospective study of children age <18 years old with type 1 diabetes followed during a 6-year period between January 1, 2018 and December 31, 2023 in the Diabetes Program at Boston Children’s Hospital (BCH), a large, pediatric, tertiary care, academic medical center. This Diabetes Program manages approximately 250 children with new-onset diabetes per year and cares for a sample of approximately 2200 total patients with type 1 diabetes.

Data were arranged in yearly cohorts from 2018 to 2023. Within each cohort, the exclusion criteria were patients with non-type 1 diabetes and diabetes duration <1 year, as well as those who transferred into the BCH Diabetes Program less than a year prior to their last endocrinologist/diabetes nurse educator (DNE) encounter in that calendar year and those who do not receive their routine diabetes care at BCH (e.g., patients seen for second opinions, consultations for non-diabetes concerns, and international patients who only have periodic diabetes follow-up at BCH). Ethics approval was granted by Boston Children’s Hospital Institutional Review Board. A waiver of informed consent was obtained.

### 2.2. Collection of variables

Eligible patients with type 1 diabetes were identified from an Outpatient Diabetes Population Management database used for quality improvement, as well as review of the electronic medical record (EMR). In each calendar year, data were collected from the final endocrinologist/DNE encounter (excluding group visits and appointments exclusively for initiation of technologies) before the end of that year or the patient’s 18th birthday.

Sociodemographic variables (as documented in the EMR) included date of birth, sex, race and ethnicity, primary language, need for interpreter, primary insurance type, and zip code. For race and ethnicity, the following categories were included: 1) White, non-Hispanic, 2) Black, non-Hispanic, 3) Hispanic, 4) Asian, non-Hispanic, 5) Multiracial, non-Hispanic, and 6) Another, non-Hispanic. Individuals whose race and ethnicity were listed as “Unknown” or “Declined to Answer” (3.4 % of total cases) were considered as “missing.” Primary language was categorized as 1) English, 2) Spanish, or 3) other. Primary insurance type was categorized as either private or public using definitions applied by the BCH EMR (those without insurance were classified under public insurance and those who chose self-pay were classified under private insurance).

Zip codes were linked to Child Opportunity Index (COI) data [30,31]. The COI is a composite index of children’s neighborhood-level opportunity that contains annual data for every neighborhood in the US between 2012 and 2021 and is comprised of 44 neighborhood indicators spanning three domains (education, health and environment, and social and economic) and 14 subdomains. The COI score ranks US neighborhoods based on the opportunities they provide for children and ranges from 1 (lowest) to 100 (highest). For this study, zip code-level COI 3.0 estimates were used. These estimates were calculated as weighted averages of 2010 COI 3.0 census tract data (i.e., census tracts that are home to a greater proportion of addresses with a given zip code receive greater weight) and were time-varying (annual data available from 2012 to 2021) [30,31]. For 2022 and 2023 patient data, the 2021 estimates were used, under the assumption that these would not have changed substantially over the course of the subsequent 1–2 years.

Diabetes care-related variables at the last endocrinologist/DNE encounter for each calendar year included the date of the most recent visit with a registered dietitian (RD) that preceded the encounter; diabetes duration, defined as the time between the date when the diagnosis code for “type 1 diabetes” (ICD-9 codes 250.X1 or 250.X3, where X = 0–9, or ICD-10 codes E10.X) first appeared in the EMR (confirmed/updated based on EMR information) and the date of the encounter; use of a continuous glucose monitor (CGM); and mode of insulin delivery (multiple daily injections, insulin pump without automated insulin delivery, or HCL system).

Additional clinical variables at the last endocrinologist/DNE encounter for each calendar year included hemoglobin A1c (HbA1c) and BMI z-score. For missing data, EMR review was conducted to find the most proximal value, provided that it was within 1 year preceding their last endocrinologist/DNE encounter. If both point-of-care testing HbA1c and whole blood HbA1c results were available on the same day, only the whole blood HbA1c value was included. Ranges for both measurements are the same, and both methods are certified by the National Glycohemoglobin Standardization Program and performed under the requisite Clinical Laboratory Improvement Amendment standards as recommended by the ADA [32]. For weight classification, patients were classified as having overweight or obesity based on their BMI z-score.

### 2.3. Statistical analyses

Summary statistics for each year were presented as observed means and standard deviations or percentages, as appropriate. To account for repeated measures, generalized estimating equations (GEE) with robust standard errors (SEs) clustering at the patient level were conducted to assess for differences in the rates of CGM use and mode of insulin delivery (controlling for age, sex, and diabetes duration) and HbA1c and BMI z-score (controlling for age, sex, diabetes duration, CGM use, and mode of insulin delivery) in years 2019–2023 compared with 2018. Additional analyses comparing specific years were performed as necessary.

GEE with robust SEs clustering at the patient level were also conducted to assess for differences over time in the rates of overweight and obesity for the whole sample and in the rates of obesity for groups subset by mode of insulin delivery, as well as to identify potential sociodemographic and diabetes care-related characteristics associated with obesity. Multivariable analysis was conducted for the latter and included calendar year as a categorical covariate to account for potential time-related confounding, including concomitant increases in the both the predictor and outcome variables and the impact of the COVID-19 pandemic. For significant categorical covariates, adjusted prevalences of obesity were reported for each category of that variable, while keeping all the covariates from the multivariable model constant, to quantify the observed effects.

As a secondary analysis, conditional logistic regression (CLR) with matched groups at the patient level was conducted among patients who had at least one BMI z-score not consistent with obesity and at least one that met criteria for obesity to assess the relationship between independent predictor variables and the outcome (obesity). As this is a within-subjects analysis, where each patient has both control status (not obesity) and case status (obesity), we excluded predictor variables that are not expected to vary within person (i.e., sex, race and ethnicity, primary language, and need for interpreter). In addition, since we included calendar year as a covariate, we also excluded variables that, within subjects, would be collinear with year (i.e., age and diabetes duration). The association of these excluded variables with obesity can be seen in the results for the population-averaged GEE model.

A two-sided *P* value of <0.05 defined statistical significance. Stata Special Edition software package (Version 16.1, StataCorp LLC, College Station, TX) was used for statistical analyses.

## 3. Results

A total of 7360 observations among 2242 unique patients followed by the BCH Diabetes Program between 2018 and 2023 were included in the analysis. Sociodemographic and diabetes care-related patient characteristics by year are summarized in Table 1. The observed percentages of patients who used a CGM increased across all 6 years, with the greatest increase from 2018 to 2019 (52.3 % to 71.3 %) and ongoing increases through the final two-year interval 2022–2023 (89.9 % to 91.1 %). Use of an HCL system increased steadily after 2019, with the largest increase from 2022 to 2023 (36.8 % to 62.8 %). In analysis adjusted for repeated measures and controlled for age, sex, and diabetes duration, HbA1c was significantly lower in 2021 and 2022 compared with 2018. There was a modest increase in HbA1c between 2022 and 2023 (*P* < 0.001). There was a gradual rise in BMI z-score (all years differed significantly from 2018) in analysis adjusted for repeated measures and controlled for age, sex, and diabetes duration.

**Fig. 1.**
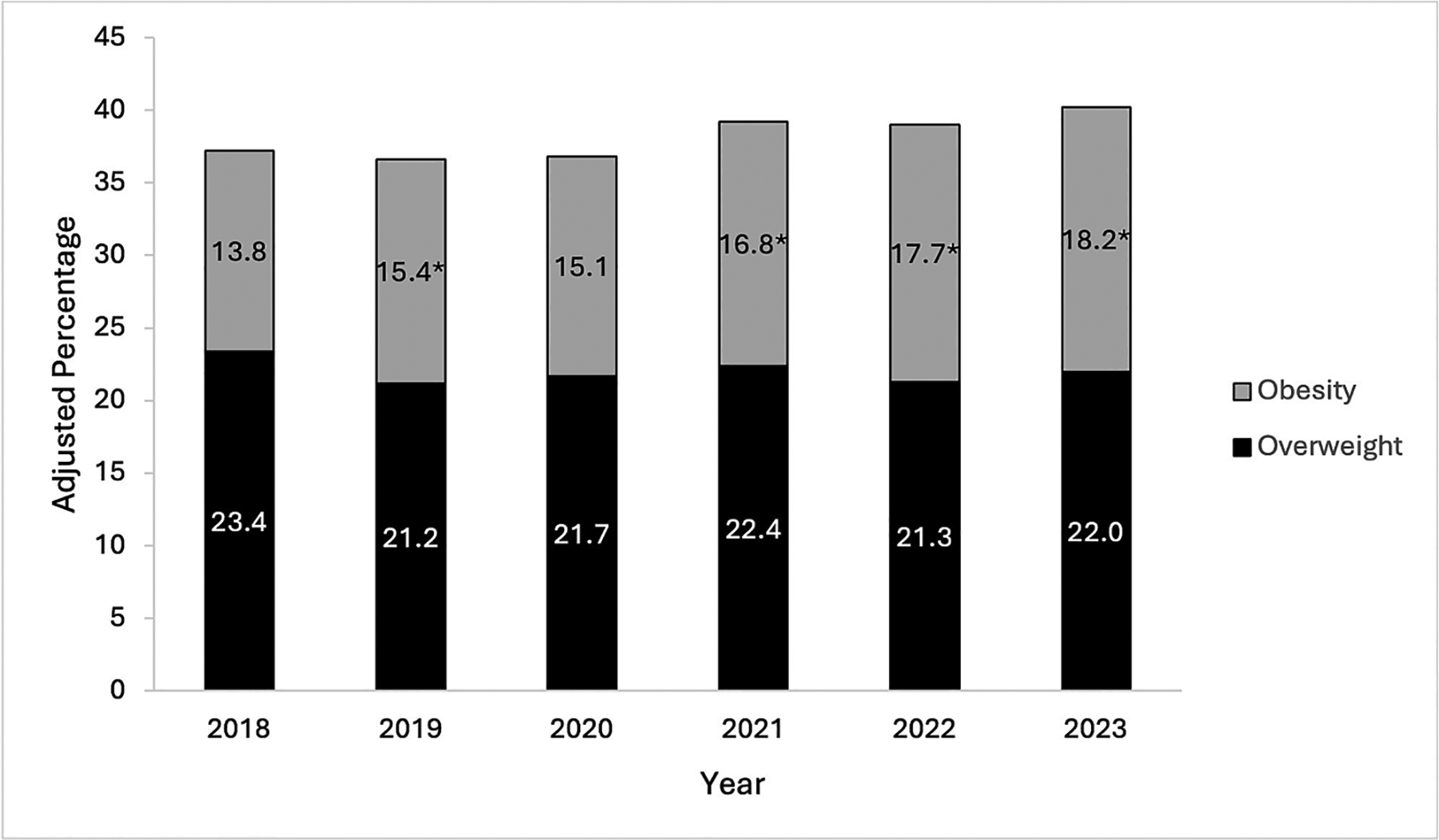
Adjusted Percentages of Established Pediatric Patients with Type 1 Diabetes Meeting Criteria for Overweight and Obesity over Time. Stack bar chart depicting adjusted annual percentages of established pediatric patients with type 1 diabetes who met criteria for overweight and obesity between 2018 and 2023. * Rates of obesity in these years were significantly different compared with 2018.

Percentages of patients meeting criteria for overweight and obesity over time, after accounting for repeated measures, are shown in Fig. 1. There were no differences in the rates of overweight compared with 2018. The rates of obesity differed over time (*P* = 0.006). Compared with 2018, rates were significantly higher in 2019, 2021, 2022, and 2023 (*P* < 0.05 for each).

In multivariable GEE analysis, public insurance (OR 1.28 [1.03–1.58; *P* = 0.026]), a lower COI score (OR 0.99 [0.99–1.00; *P* = 0.027]), and HCL system use (OR 1.27 [1.03–1.56; *P* = 0.023]) were associated with obesity (Table 2). In addition, patients in 2019 (OR 1.15 [1.02–1.31; *P* = 0.024]), 2021 (OR 1.29 [1.08–1.54; *P* = 0.005]), 2022 (OR 1.32 [1.10–1.59; *P* = 0.004]), and 2023 (OR 1.31 [1.06–1.62; *P* = 0.013]) had significantly higher odds of obesity compared with 2018. Adjusted prevalences of obesity in each category of the 3 significant categorical predictors are depicted in Supplemental Table 1 (all other variables from the multivariable model were held constant during the analyses).

In CLR of factors associated with obesity among a nested sample of patients who had at least one BMI z-score not consistent with obesity and at least one that met criteria for obesity, there were a total of 946 observations (among 213 unique patients) (Table 3). In this within-subjects analysis, use of CGM (OR 1.95 [1.12–3.37; *P* = 0.017]) and HCL systems (OR 3.16 [1.56–6.40; *P* = 0.001]) were significantly associated with obesity, while primary insurance type and COI score were not. The sum of CGM and HCL system effects resulted in OR 3.56 (1.57–8.09; *P* = 0.002). Only the years 2021 (OR 2.01 [1.12–3.59; *P* = 0.019]) and 2022 (OR 1.85 [1.01–3.38; *P* = 0.046]) were associated with higher odds of obesity compared with 2018.

**Fig. 2.**
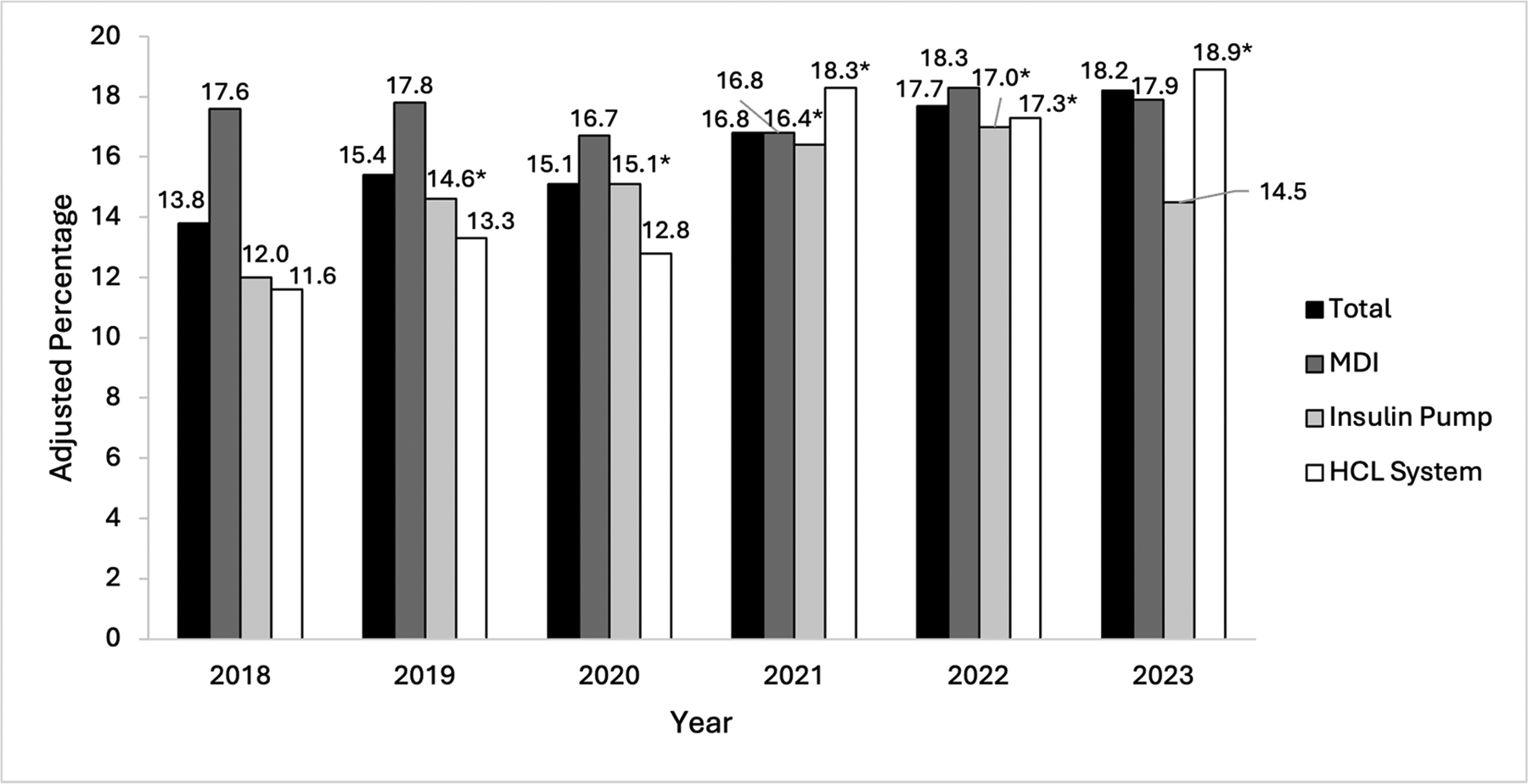
Adjusted Percentages of Established Pediatric Patients with Type 1 Diabetes Meeting Criteria for Obesity over Time Subset by Mode of Insulin Delivery. Bar chart depicting adjusted annual percentages of established pediatric patients with type 1 diabetes who met criteria for obesity between 2018 and 2023 subset by mode of insulin delivery. * Rates of obesity in these years were significantly different compared with 2018. *Abbreviations:* HCL, hybrid closed-loop; MDI, multiple daily injections.

Percentages of patients meeting criteria for obesity over time when subset by mode of insulin delivery and after accounting for repeated measures are shown in Fig. 2. MDI therapy was not associated with significant differences in the rates of obesity compared with 2018. Insulin pump therapy (without automated insulin delivery) was associated with significantly higher rates of obesity in 2019, 2020, and 2022 compared with 2018 (*P* < 0.05 for each). HCL system use was associated with significantly higher rates of obesity in 2021, 2022, and 2023 compared with 2018 (*P* < 0.05 for each). The overall increase in the prevalence of obesity was the highest among patients using HCL systems (11.6 % in 2018 to 18.9 % in 2023).

## 4. Discussion

This study found significant increases in the rates of obesity over time among established pediatric patients with type 1 diabetes followed in a pediatric, tertiary care, academic medical center. The percentage of patients with obesity in this population as of 2023 (18.2 %), after accounting for repeated measures, was lower than the most recently available national prevalence of pediatric obesity in the US (22.4 % as of August 2020) [1]. The percentages of overweight, after accounting for repeated measures, have remained largely unchanged among established pediatric patients with type 1 diabetes at our institution; however, the rates of either overweight or obesity in this dynamic cohort increased from 37.2 % in 2018 to 40.2 % in 2023. This appears to be similar to the most recently available data on all youth in the US from 2021, estimating the prevalence of overweight and obesity ranging from 21.1 % in boys aged 2–4 years up to 45.6 % in adolescent girls aged 15–19 years [2]. It is clear that the rates of obesity are increasing over time in all youth, including children with type 1 diabetes, a finding supported by our multivariable GEE analysis and CLR both showing significant associations between calendar year and obesity. The effect of the COVID-19 pandemic, with its associated preventive measures and changes in eating habits and activity levels [33], is also suggested by significantly higher odds of having obesity in 2021, 2022, and 2023 compared to 2018. However, our study found additional sociodemographic and diabetes care-related factors associated with a higher likelihood of obesity in youth with type 1 diabetes that warrant further consideration.

Among demographic factors, our study identified public insurance and lower COI score (suggesting less favorable neighborhood characteristics [30,31]) as being associated with obesity in children with type 1 diabetes in multivariable GEE analysis, even when accounting for potential time-related confounding (including the effect of the COVID-19 pandemic). Of note, COI score was also recently associated with a higher risk of obesity specifically among black youth with type 1 diabetes at the time of diagnosis [16]. Public insurance and COI score were not significantly associated with obesity in CLR, likely because this is a within-subject analysis (as opposed to the GEE, which is a between-subject analysis). In this analysis, the associations are conditional on the groups (i.e., patients in this case), so if there is little variation within groups, which we would expect for factors such as insurance status and COI score, then there is not enough power to find associations.

Dependence on public insurance and a lower COI score suggest potential sociodemographic inequities with respect to access to healthier food options and opportunities for physical activity, thus predisposing these patients to excessive weight gain. It is notable that in our study, use of public insurance increased from 23.6 % in 2018 to 32.5 % in 2023, potentially placing greater health risks among a larger group of pediatric patients with type 1 diabetes over time. In patients with type 2 diabetes, the presence of severe food insecurity has also been associated with higher BMI in the setting of lower overall caloric consumption but intake of energy-dense foods with higher glycemic load [34]. There is a paucity of data on this topic in type 1 diabetes [35], but it deserves further study. Potential targets for future intervention may include providing more nutrition education for those with public insurance or other adverse socioeconomic characteristics and offering social service support for those families of youth with type 1 diabetes living with food insecurity.

Among diabetes care-related factors, we observed a significant association between the use of HCL systems and obesity in pediatric patients with type 1 diabetes in both multivariable GEE analysis and CLR. Patients using these technologies demonstrated substantial increases in the prevalence of obesity over time (11.6 % in 2018 to 18.9 % in 2023), after accounting for repeated measures (Fig. 2). Although the rates of both HCL system use and obesity increased over time in our cohort, their association persisted even when accounting for potential time-related confounding. It is also notable that HCL system use was significantly associated with obesity in CLR. The use of a CGM system was also significantly associated with obesity in CLR. Since, by definition, everyone on an HCL system uses a CGM, then the true risk of obesity associated with HCL systems is the sum of the CGM and HCL system effects (OR 3.56 [1.57–8.09]; *P* = 0.002). Although this analysis does not establish the directionality of the association, there is no clinical reason to suspect that patients with obesity were more likely to go on HCL systems than those who did not have obesity.

The use of HCL systems has increased significantly over the last several years, an observation supported by our study. This technology has revolutionized contemporary diabetes management as demonstrated by improvements in glycemic control, enhanced quality of life, and decreased disease burden in patients with type 1 diabetes across the lifespan [21–23,25–27]. The effect of HCL systems on weight in patients with type 1 diabetes has been inconclusive, with some short-term studies showing modest increases, especially in adults [36] and those with initial suboptimal glycemic control [37], and others not finding a significant association [23,38–40], at least in children [36]. Furthermore, the association between intensive insulin management and weight gain has been supported by many [17–19,41–44], but not all [9,28,36,38–40,45,46], prior studies, including the sentinel DCCT [17–19,41,42], performed in an era prior to availability of more physiologic insulin analogs that have been associated with reduced occurrence of hypoglycemia [20]. HCL systems can automatically increase insulin delivery in response to glucose trends, with several studies showing significantly higher total daily doses of insulin associated with their use compared to baseline [23,36–38]. Because insulin is an anabolic hormone, excessive weight gain in patients using these technologies may be at least partially attributable to the increased insulin delivery in response to glucose trends aiming to restore blood glucose to specific glycemic targets [29], although this requires further investigation.

Importantly, given that HCL systems can ameliorate postprandial glycemic excursions via increased automated insulin delivery, individuals with diabetes may perceive more freedom in food choices and permission to eat less healthfully, combined with relaxed prandial management practices, including suboptimal insulin timing and imprecise carbohydrate/insulin matching. This has been a theme in previous qualitative analysis [28] and was supported by a recent finding of decreased user-initiated boluses and carbohydrate entries within the first 90 days of HCL system use in youth with type 1 diabetes [24]. Another study did not show a significant impact of HCL system initiation on eating habits in patients with type 1 diabetes after 1 year; however, this study was performed in adults (mean age 40.9 years) [40], and recall and/or social desirability biases cannot be ruled out.

In addition, while overall time below target range may be improved with HCL system use [21,23–26], suboptimal carbohydrate/insulin matching can lead to glycemic excursions characterized by rising blood glucoses followed by temporary rebound hypoglycemia from resultant automated delivery of high insulin doses. This then requires increased carbohydrate intake, contributing to excessive weight gain. Importantly, there is potential for persons with type 1 diabetes using HCL systems to overtreat hypoglycemia despite presumed education and training at initiation of HCL systems to treat frank or impending hypoglycemia with only 5–10 g of carbohydrates due to the automated systems already attenuating or halting insulin delivery in anticipation of the low glucose [47]. It is likely challenging for people living with type 1 diabetes to alter their previous habits of treating hypoglycemia with 15–20 g of carbohydrates, thereby leading to overtreatment of lows, which then results in a rebound hyperglycemia and insulin augmentation for the ingestion of unnecessary carbohydrate intake. Thus, a potential target for intervention to prevent excessive weight gain would be reinforcement of HCL education around hypoglycemia management. Nonetheless, excessive caloric intake in general is likely a significant contributor to the association seen between HCL system use and excessive weight gain and deserves further attention.

This study has several limitations that should be noted. It was retrospective in nature, relying predominantly on EMR data. EMR data may be inaccurate, incomplete, or transformed in ways that may impact its interpretation and utility [48]. However, our use of EMR data allowed us to objectively examine the records of a large sample of patients and collect data reflecting real-world circumstances that was less prone to selection and recall biases common to studies that rely solely on self-reported information. As retrospective studies do not afford the opportunity to establish causation, future prospective studies are necessary to longitudinally examine BMI trends in children with type 1 diabetes and to identify potential factors contributing to a larger rise in BMI over time.

In addition, our study was limited to data collected from one pediatric, academic medical center, resulting in some potential limitation in external generalizability. Although BCH serves a diverse population of children with type 1 diabetes, our sample may not be representative of all youth with diabetes on a national level. In addition, diabetes education and management strategies may vary across institutions, and this may impact their obesity trends and associations in specific ways. The COVID-19 pandemic also presented several challenges to data collection. During 2020 and 2021, there were notably more missing data for HbA1c (7.8 % in 2020, 12.5 % in 2021) and BMI z-score (7.1 % in 2020, 15.3 % in 2021) compared to the other years, which may have impacted some of the analyses and findings, especially if in-person visits were directed to patients with greater management challenges, such as those from lower socioeconomic status groups. The latter groups may have reduced access to robust telehealth platforms, leading to more in-person visits and available data for the current analysis. However, in our analysis, the rate of public insurance use (used as a proxy for lower socioeconomic status) among patients with missing HbA1c (28.2 %) and BMI z-score (23.2 %) data was not, on average, significantly higher than what was observed for our whole sample (26.8 %). Lastly, since many medical visits during the COVID-19 pandemic were converted to telehealth, some of the BMI z-score values extracted from the EMR may have been based on self-reported height and weight measurements, the accuracy of which could not be verified. The Division of Endocrinology at BCH tried to mitigate this by providing families with a handout on standardized ways to perform these measurements at home prior to telehealth clinic visits.

It is important to note that recently, the Lancet Diabetes & Endocrinology Commission came out with a statement discussing limitations of using BMI to diagnose obesity and proposed a new diagnostic approach that focuses on other measures of adiposity and objective signs and symptoms of illness [49]. The Commission distinguished between preclinical and clinical obesity, with the latter characterized by ongoing organ dysfunction and/or reduced ability to conduct daily activities. The European Association for the Study of Obesity subsequently released a response to the Commission’s recommendations, raising concerns and highlighting potential risks to obesity management and patient care [50]. It is not yet clear how these discussions may affect the conceptualization and management of obesity or its long-term risks in both adults and children. Further studies are needed to understand how these new concepts may impact our approach to youth with type 1 diabetes and obesity.

In conclusion, our study showed increasing rates of obesity over a six-year period from 2018 to 2023, which overlapped with the height of the COVID-19 pandemic, among established patients with type 1 diabetes at a large, pediatric, academic medical center and identified several factors associated with a higher likelihood of obesity, including dependence on public insurance, lower COI score, and use of HCL systems. HCL systems have redefined type 1 diabetes care in recent years with immense glycemic and psychosocial benefits in this population. However, as discussed, the use of HCL systems may facilitate the development of obesity if combined with suboptimal dietary practices, imprecise prandial diabetes management behaviors, improper management of hypoglycemia, and, in some cases, limited access to healthy food choices and opportunities for physical activity, offering direction for prospective studies with a large-scale collaboration. Ultimately, insights from these studies could be used to design interventional strategies to attenuate the risk of accelerated weight gain and development of obesity in pediatric patients with type 1 diabetes.

## Supplementary Material

MMC1

## Figures and Tables

**Table 1:** Characteristics of Established Pediatric Patients with Type 1 Diabetes by Year.

Characteristic	Data by Year (Observed Mean ± SD or Percentage)					
	2018 (n = 1206)	2019 (n = 1193)	2020 (n = 1181)	2021 (n = 1226)	2022 (n = 1228)	2023 (n = 1326)
Sociodemographic Characteristics						
Age (years)	13.7 ± 3.5	13.7 ± 3.6	13.6 ± 3.7	13.5 ± 3.7	13.4 ± 3.7	13.2 ± 3.8
Female sex	48.8 %	48.6 %	50.0 %	49.3 %	47.3 %	47.6 %
Race and ethnicity*^†^						
*White, non-Hispanic*	79.7 %	78.8 %	77.9 %	77.1 %	76.3 %	73.3 %
*Black, non-Hispanic*	4.4 %	4.4 %	4.0 %	4.9 %	4.8 %	5.4 %
*Hispanic*	9.0 %	9.6 %	9.9 %	10.0 %	10.7 %	12.8 %
*Asian, non-Hispanic*	1.4 %	1.3 %	1.4 %	1.4 %	1.4 %	2.0 %
*Multiracial, non-Hispanic*	1.2 %	1.5 %	1.7 %	1.8 %	2.1 %	2.0 %
*Another, non-Hispanic*	4.4 %	4.4 %	5.1 %	4.9 %	4.8 %	4.5 %
Primary language^†^						
*English*	96.6 %	96.6 %	96.0 %	95.7 %	95.6 %	94.6 %
*Spanish*	1.7 %	1.8 %	2.1 %	2.2 %	2.3 %	2.6 %
*Other*	1.7 %	1.6 %	1.9 %	2.1 %	2.1 %	2.9 %
Need for interpreter	2.4 %	2.8 %	3.0 %	3.2 %	3.5 %	4.3 %
Public insurance	23.6 %	24.0 %	24.8 %	26.9 %	28.8 %	32.5 %
COI score	80 ± 22	81 ± 22	81 ± 22	81 ± 21	81 ± 22	79 ± 23
Living in low-income zip codes*	24.5 %	25.1 %	24.7 %	25.1 %	24.5 %	25.0 %
Living in zip codes with lower educational attainment*	25.0 %	24.7 %	25.0 %	25.0 %	25.6 %	24.1 %
Diabetes Care-related Characteristics						
Diabetes duration (years)	6.0 ± 3.7	5.9 ± 3.7	5.7 ± 3.7	5.6 ± 3.6	5.5 ± 3.4	5.5 ± 3.4
CGM use^‡^	52.3 %	71.3 %^§^	78.4 %^§^	84.7 %^§^	89.9 %^§^	91.1 %^§^
Mode of insulin delivery^†,‡^						
*MDI*	38.9 %	38.2 %^§^	39.7 %	41.0 %	36.9 %^§^	29.3 %^§^
*Insulin pump (without automation)*	58.0 %	58.4 %	48.4 %^§^	41.0 %^§^	26.3 %^§^	7.9 %^§^
*HCL system*	3.1 %	3.4 %	11.9 %^§^	17.9 %^§^	36.8 %^§^	62.8 %^§^
Hemoglobin A1c (%)^||^ [mmol/mol]	8.5 ± 1.5 [68.9 ± 16.1]	8.3 ± 1.6 [66.9 ± 17.5]	8.2 ± 1.6 [66.5 ± 17.4]	7.9 ± 1.6^§^ [63.2 ± 17.8]	7.8 ± 1.6^§^ [61.3 ± 17.6]	8.0 ± 1.6 [63.5 ± 17.1]
BMI z-score^||^	0.63 ± 0.94	0.65 ± 0.95^§^	0.69 ± 0.95^§^	0.70 ± 0.95^§^	0.71 ± 0.96^§^	0.73 ± 0.95^§^
RD visit in the preceding year	20.6 %	19.4 %	21.4 %	20.3 %	22.5 %	22.8 %

*Abbreviations:* BMI, body mass index; CGM, continuous glucose monitor; COI, Child Opportunity Index; HCL, hybrid closed-loop; MDI, multiple daily injections; RD, registered dietitian; SD, standard deviation.

* Missing data were not included in the denominator.

† Totals may not sum up to 100 % due to rounding.

‡ Although observed values are reported, comparative analyses across years were adjusted for repeated measures and controlled for age, sex, and diabetes duration.

§ Values in these years differed significantly from 2018, *P* < 0.05.

|| Although observed values are reported, comparative analyses across years were adjusted for repeated measures and controlled for age, sex, diabetes duration, CGM use, and mode of insulin delivery.

**Table 2:** Multivariable Analysis of Factors Associated with Obesity in 2242 Children with Type 1 Diabetes.

Characteristic	OR (95 % CI)	Robust SE	z	*P* value
Age (years)	1.01 (0.98 to 1.04)	0.02	0.68	0.495
Sex				
*Male*	REF	REF	REF	REF
*Female*	1.06 (0.86 to 1.32)	0.12	0.57	0.566
Race and ethnicity				
*White, non-Hispanic*	REF	REF	REF	REF
*Black, non-Hispanic*	1.17 (0.71 to 1.94)	0.30	0.62	0.538
*Hispanic*	1.29 (0.88 to 1.88)	0.25	1.31	0.190
*Asian, non-Hispanic*	1.14 (0.55 to 2.37)	0.43	0.35	0.727
*Multiracial, non-Hispanic*	1.02 (0.46 to 2.29)	0.42	0.05	0.959
*Another, non-Hispanic*	0.91 (0.58 to 1.42)	0.21	−0.41	0.681
Primary language				
*English*	REF	REF	REF	REF
*Spanish*	1.19 (0.49 to 2.89)	0.54	0.39	0.695
*Other*	0.86 (0.37 to 2.02)	0.37	−0.34	0.733
Need for interpreter				
*No*	REF	REF	REF	REF
*Yes*	0.74 (0.31 to 1.73)	0.32	−0.71	0.481
Primary insurance type				
*Private*	REF	REF	REF	REF
*Public*	1.28 (1.03 to 1.58)	0.14	2.22	**0.026**
COI score	0.99 (0.99 to 1.00)	0.00	−2.21	**0.027**
Diabetes duration (years)	0.98 (0.95 to 1.01)	0.02	−1.45	0.147
CGM use				
*No*	REF	REF	REF	REF
*Yes*	1.08 (0.92 to 1.28)	0.09	0.95	0.343
Mode of insulin delivery				
*MDI*	REF	REF	REF	REF
*Insulin pump*	1.04 (0.87 to 1.26)	0.10	0.47	0.642
*HCL system*	1.27 (1.03 to 1.56)	0.13	2.27	**0.023**
Hemoglobin A1c (%) [mmol/mol]	1.01 (0.96 to 1.06) [1.00 (1.00 to 1.00)]	0.02 [0.00]	0.31	0.755
RD visit in the preceding year	1.12 (0.99 to 1.27)	0.07	1.84	0.066
Calendar year				
*2018*	REF	REF	REF	REF
*2019*	1.15 (1.02 to 1.31)	0.07	2.26	**0.024**
*2020*	1.12 (0.96 to 1.32)	0.09	1.47	0.141
*2021*	1.29 (1.08 to 1.54)	0.12	2.80	**0.005**
*2022*	1.32 (1.10 to 1.59)	0.13	2.92	**0.004**
*2023*	1.31 (1.06 to 1.62)	0.14	2.48	**0.013**

*Abbreviations*: CGM, continuous glucose monitor; CI, confidence interval; COI, Child Opportunity Index; HCL, hybrid closed-loop; MDI, multiple daily injections; OR, odds ratio; RD, registered dietitian; REF, reference; SE, standard error.

**Table 3:** Conditional Logistic Regression of Factors Associated with Obesity Among 213 Children with Type 1 Diabetes.

Characteristic	OR (95 % CI)	Robust SE	z	*P* value
Public insurance type	1.36 (0.59 to 3.11)	0.57	0.72	0.472
COI score	0.92 (0.85 to 1.00)	0.04	−1.96	0.050
CGM use				
*No*	REF	REF	REF	REF
*Yes*	1.95 (1.12 to 3.37)	0.54	2.38	**0.017**
Mode of insulin delivery				
*MDI*	REF	REF	REF	REF
*Insulin pump*	1.83 (1.00 to 3.36)	0.57	1.85	0.051
*HCL system*	3.16 (1.56 to 6.40)	1.14	3.20	**0.001**
Hemoglobin A1c (%) [mmol/mol]	0.85 (0.71 to 1.01) [0.98 (0.97 to 1.00)]	0.08 [0.01]	−1.85	0.065
RD visit in the preceding year	1.23 (0.83 to 1.84)	0.25	1.03	0.303
Calendar year				
*2018*	REF	REF	REF	REF
*2019*	1.64 (0.99 to 2.71)	0.42	1.93	0.053
*2020*	1.47 (0.86 to 2.50)	0.40	1.42	0.157
*2021*	2.01 (1.12 to 3.59)	0.59	2.35	**0.019**
*2022*	1.85 (1.01 to 3.38)	0.57	1.99	**0.046**
*2023*	1.82 (0.92 to 3.59)	0.63	1.71	0.085

*Abbreviations*: CGM, continuous glucose monitor; CI, confidence interval; COI, Child Opportunity Index; HCL, hybrid closed-loop; MDI, multiple daily injections; OR, odds ratio; RD, registered dietitian; REF, reference; SE, standard error.

## Data Availability

The datasets generated and analyzed in the current study are available from the corresponding author upon reasonable request.
