# Supplementary material for: Sociodemographic inequities and use of hybrid closed-loop systems associated with obesity in youth with type 1 diabetes
Source: Diabetes Res Clin Pract. Author manuscript; Available in PMC 2025 Apr 16. (PMC11913344; doi:10.1016/j.diabres.2025.112041)
Supplement: MMC1 [file NIHMS2058460-supplement-MMC1.docx]

**Supplemental Table 1. Adjusted Prevalences of Obesity in Children with Type 1 Diabetes by Category of Significant Predictors from Multivariable Analysis**

| **Variable*** | **Adjusted Prevalence** |
| --- | --- |
| Primary insurance type |  |
| *Private* | 15.6% |
| *Public* | 19.0% |
| Mode of insulin delivery |  |
| *MDI* | 15.6% |
| *Insulin pump* | 16.2% |
| *HCL system* | 18.9% |
| Calendar year |  |
| *2018* | 14.2% |
| *2019* | 16.0% |
| *2020* | 15.7% |
| *2021* | 17.6% |
| *2022* | 17.9% |
| *2023* | 17.7% |

* All other variables from the multivariable model were held constant during the analyses.

*Abbreviations:* HCL, hybrid closed-loop; MDI, multiple daily injections.
